# Supplementary material for: Prediction of clinical deterioration within one year in chronic obstructive pulmonary disease using the systemic coagulation-inflammation index: a retrospective study employing multiple machine learning method
Source: PeerJ. 2025 Feb 25;13:e18989. doi: 10.7717/peerj.18989 (PMC11869895; doi:10.7717/peerj.18989)
Supplement: Supplemental Information 1 [file peerj-13-18989-s001.docx]

STROBE Statement—checklist of items that should be included in reports of observational studies

|  | Item No. | Recommendation | Page  No. | Relevant text from manuscript |
| --- | --- | --- | --- | --- |
| **Title and abstract** | 1 | (*a*) Indicate the study’s design with a commonly used term in the title or the abstract | 1 | A Retrospective Study |
|  |  | (*b*) Provide in the abstract an informative and balanced summary of what was done and what was found | 2 | The systemic coagulation-inflammation (SCI) index is associated with clinical deterioration in COPD. |
| Introduction | | | |  |
| Background/rationale | 2 | Explain the scientific background and rationale for the investigation being reported | 3 | COPD is a progressive respiratory condition characterized by irreversible airflow limitation^1^. The escalating global incidence and associated mortality rates of COPD present a significant challenge to the healthcare industry worldwide.The clinical deterioration of COPD is linked to escalated systemic and airway inflammatory responses, with increased inflammation exacerbating clinical symptoms and impairing lung function in individuals^6^ |
| Objectives | 3 | State specific objectives, including any prespecified hypotheses | 3 | The systemic coagulation-inflammation index, an innovative hematological marker, effectively mirrors the dynamics of coagulation disorders and inflammation. Thus, we hypothesize a correlation between cd-COPD occurrence and SCI levels. |
| Methods | | | |  |
| Study design | 4 | Present key elements of study design early in the paper | 3 | The data was collected from January 2018 to December 2021 on COPD diagnosed patients admitted to our hospital, identified by International Classification of Diseases, 10th Revision (ICD-10) codes J440, J441, J449. |
| Setting | 5 | Describe the setting, locations, and relevant dates, including periods of recruitment, exposure, follow-up, and data collection | 3 |  |
| Participants | 6 | (*a*) *Cohort study*—Give the eligibility criteria, and the sources and methods of selection of participants. Describe methods of follow-up  *Case-control study*—Give the eligibility criteria, and the sources and methods of case ascertainment and control selection. Give the rationale for the choice of cases and controls  *Cross-sectional study*—Give the eligibility criteria, and the sources and methods of selection of participants | 3 | Clinical deterioration was defined as at least one acute exacerbation event requiring hospitalization within one year after discharge. Patients were ultimately categorized into the cd-COPD group and non-cd-COPD group. Exclusion criteria included severe immune, or psychiatric illnesses; presence of malignancies, hematologic disorders, or hepatic/renal dysfunction; and loss to follow-up |
|  |  | (*b*) *Cohort study*—For matched studies, give matching criteria and number of exposed and unexposed  *Case-control study*—For matched studies, give matching criteria and the number of controls per case |  |  |
| Variables | 7 | Clearly define all outcomes, exposures, predictors, potential confounders, and effect modifiers. Give diagnostic criteria, if applicable | 4 | Clinical data, encompassing demographic details (gender, age), comorbidities (e.g., hypertension, diabetes, coronary heart disease), and various laboratory parameters (complete blood count, random venous glucose, lipid profile), were extracted from the electronic medical records. Treatment details during hospitalization, such as mechanical ventilation and length of stay for initial COPD diagnosis, were also included. |
| Data sources/ measurement | 8* | For each variable of interest, give sources of data and details of methods of assessment (measurement). Describe comparability of assessment methods if there is more than one group | *4* | The Systemic Coagulation-Inflammation Index was calculated using the formula: (platelet count * fibrinogen) / white blood cell count upon admission |
| Bias | 9 | Describe any efforts to address potential sources of bias | 3 | The data was collected from January 2018 to December 2021 on COPD diagnosed patients admitted to our hospital, identified by International Classification of Diseases, 10th Revision (ICD-10) codes J440, J441, J449 |
| Study size | 10 | Explain how the study size was arrived at | 3 | The data was collected from January 2018 to December 2021 on COPD diagnosed patients admitted to our hospital, identified by International Classification of Diseases, 10th Revision (ICD-10) codes J440, J441, J449 |

Continued on next page

| Quantitative variables | 11 | Explain how quantitative variables were handled in the analyses. If applicable, describe which groupings were chosen and why | 4 | Skewed data were described using the interquartile range (IQR) and assessed using the Mann-Whitney U test. |
| --- | --- | --- | --- | --- |
| Statistical methods | 12 | (*a*) Describe all statistical methods, including those used to control for confounding | 4 | Statistical analysis was conducted using R 4.3.2 and python 3.9. Student’s t-test was utilized for normally distributed continuous variables, while the chi-square test or Fisher’s exact test was employed for categorical variables. Skewed data were described using the interquartile range (IQR) and assessed using the Mann-Whitney U test. Six ML algorithms were utilized, namely, XGBoost, logistic regression, random forest, Elastic Net, support vector machine, and k-nearest neighbors. Model training encompassed 70% of the samples, with the remaining 30% reserved for testing. To prevent overfitting, adjustments were made during training, and missing data were handled through KNN imputation. The optimal hyperparameters were selected via 5-fold cross-validation, and model performance was evaluated based on AUC, sensitivity, specificity, and overall accuracy. The best model was chosen, and variable importance ranking was conducted. Calibration curve analysis was performed to assess model fitting, comparing predicted and true probabilities visually. To delve deeper into the SVM model, the Shapley Additive Explanations (SHAP) method is employed to assess the significance of various predictive variables on the model’s predictive outcomes. Through computation of SHAP values and visual inspection of the model, the extent to which each variable contributes to the progression of clinical deterioration in chronic obstructive pulmonary disease over a one-year period is investigated. All statistical tests were two-tailed, with significance set at *p* < 0.05. |
|  |  | (*b*) Describe any methods used to examine subgroups and interactions | 4 |  |
|  |  | (*c*) Explain how missing data were addressed | 4 |  |
|  |  | (*d*) *Cohort study*—If applicable, explain how loss to follow-up was addressed  *Case-control study*—If applicable, explain how matching of cases and controls was addressed  *Cross-sectional study*—If applicable, describe analytical methods taking account of sampling strategy | 4 |  |
|  |  | (*e*) Describe any sensitivity analyses |  | No applicable, |
| Results | | | | |
| Participants | 13* | (a) Report numbers of individuals at each stage of study—eg numbers potentially eligible, examined for eligibility, confirmed eligible, included in the study, completing follow-up, and analysed | 3 |  |
|  |  | (b) Give reasons for non-participation at each stage | 3 |  |
|  |  | (c) Consider use of a flow diagram | 3 |  |
| Descriptive data | 14* | (a) Give characteristics of study participants (eg demographic, clinical, social) and information on exposures and potential confounders | 4 |  |
|  |  | (b) Indicate number of participants with missing data for each variable of interest | 4 |  |
|  |  | (c) *Cohort study*—Summarise follow-up time (eg, average and total amount) | 4 |  |
| Outcome data | 15* | *Cohort study*—Report numbers of outcome events or summary measures over time | *4* |  |
|  |  | *Case-control study—*Report numbers in each exposure category, or summary measures of exposure |  |  |
|  |  | *Cross-sectional study—*Report numbers of outcome events or summary measures |  |  |
| Main results | 16 | (*a*) Give unadjusted estimates and, if applicable, confounder-adjusted estimates and their precision (eg, 95% confidence interval). Make clear which confounders were adjusted for and why they were included |  | No applicable, |
|  |  | (*b*) Report category boundaries when continuous variables were categorized | Table 1 |  |
|  |  | (*c*) If relevant, consider translating estimates of relative risk into absolute risk for a meaningful time period |  | No applicable, |

Continued on next page

| Other analyses | 17 | Report other analyses done—eg analyses of subgroups and interactions, and sensitivity analyses |  | No applicable, |
| --- | --- | --- | --- | --- |
| Discussion | | | | |
| Key results | 18 | Summarise key results with reference to study objectives | 5 | In this retrospective study of 957 patients, it was found that the level of SCI was significantly higher in cd-COPD patients compared to COPD patients |
| Limitations | 19 | Discuss limitations of the study, taking into account sources of potential bias or imprecision. Discuss both direction and magnitude of any potential bias | 7 | our study utilized a single-center retrospective design, which may introduce selection bias in some samples, thereby limiting the generalizability of the study results. |
| Interpretation | 20 | Give a cautious overall interpretation of results considering objectives, limitations, multiplicity of analyses, results from similar studies, and other relevant evidence | 7 | Despite comparing various machine learning models and validating the stability and good generalization ability of the SVM model, we cannot completely rule out the potential predictive advantages of other models. Additionally, due to the limitations of machine learning models, the predictive results may be influenced by data quality and feature selection, requiring further validation and optimization in practical applications. |
| Generalisability | 21 | Discuss the generalisability (external validity) of the study results | 7 | this study only provides preliminary exploration of the relationship between SCI and COPD clinical deterioration, necessitating further in-depth research and practice. |
| Other information | |  | | |
| Funding | 22 | Give the source of funding and the role of the funders for the present study and, if applicable, for the original study on which the present article is based | 8 | This research received no specific funding from public, commercial, or non-profit sectors. |

*Give information separately for cases and controls in case-control studies and, if applicable, for exposed and unexposed groups in cohort and cross-sectional studies.

**Note:** An Explanation and Elaboration article discusses each checklist item and gives methodological background and published examples of transparent reporting. The STROBE checklist is best used in conjunction with this article (freely available on the Web sites of PLoS Medicine at http://www.plosmedicine.org/, Annals of Internal Medicine at http://www.annals.org/, and Epidemiology at http://www.epidem.com/). Information on the STROBE Initiative is available at www.strobe-statement.org.
